# Essentiality of nickel in plants: a role in plant stresses

**DOI:** 10.3389/fpls.2015.00754

**Published:** 2015-09-23

**Authors:** Caio C. Fabiano, Tiago Tezotto, José L. Favarin, Joseph C. Polacco, Paulo Mazzafera

**Affiliations:** ^1^Departamento de Biologia Vegetal, Instituto de Biologia, Universidade Estadual de CampinasCampinas, Brazil; ^2^Departamento de Produção Vegetal, Universidade de São Paulo, Escola Superior de Agricultura Luiz de QueirozPiracicaba, Brazil; ^3^Interdisciplinary Plant Group, Department of Biochemistry, University of MissouriColumbia, MO, USA

**Keywords:** nickel, oxidative stress, glutathione reductase, glyoxalase, methylglyoxal

## Abstract

The element Ni is considered an essential plant micronutrient because it acts as an activator of the enzyme urease. Recent studies have shown that Ni may activate an isoform of glyoxalase I, which performs an important step in the degradation of methylglyoxal (MG), a potent cytotoxic compound naturally produced by cellular metabolism. Reduced glutathione (GSH) is consumed and regenerated in the process of detoxification of MG, which is produced during stress (stress-induced production). We examine the role of Ni in the relationship between the MG cycle and GSH homeostasis and suggest that Ni may have a key participation in plant antioxidant metabolism, especially in stressful situations.

## Nickel, an essential micronutrient in plants

The essentiality of nickel as a micronutrient in plants has been established because it is part of the active site of the enzyme urease, which hydrolyzes urea in plant tissues (Polacco, 1977; Eskew et al., 1983, 1984). Two forms of urease are present in plants, one found in seeds and another found in vegetative tissues (ubiquitous). The seed form of urease is highly active (Polacco et al., 2013), while the activity of the ubiquitous form is low in vegetative tissues, despite playing an important role in N recycling in plants (Hogan et al., 1983). Potentially toxic amounts of urea are metabolized into ammonia by the action of ubiquitous urease, and the N from the ammonia may be reused in other metabolic pathways, e.g., synthesis of amino acids, polyamines, and other nitrogen compounds (Gerendás and Sattelmacher, 1997, 1999).

## Glyoxalases

Glyoxalase I has been extensively studied in microorganism but much less in plants, although its characterization in mono- and dicotyledonous (Kaur et al., 2013). In microorganisms glyoxalase I may requires Ni(II) or Zn(II) for activity but only recently it was shown in rice (*Oryza sativa*) that Ni may also activate this enzyme in plants (Mustafiz et al., 2014). Studies tracing the origin of metal ion requirement of glyoxalase I in plants suggested that gene expansion led to multiple two-domain Ni-Glyoxalase I and different forms of the enzyme have evolved to help plants adapt to stress (Kaur et al., 2013).

**Figure 1:**
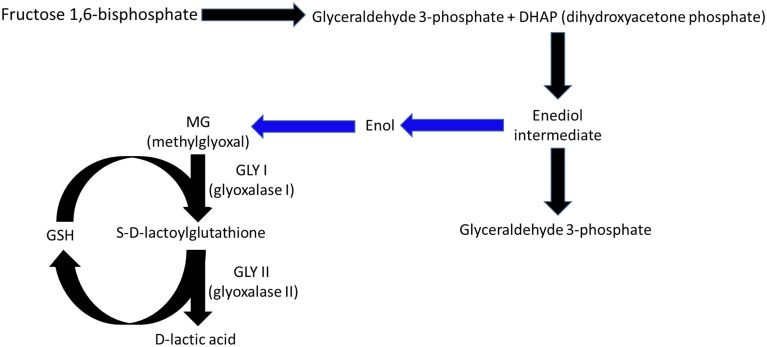
**Under normal physiological conditions, methylglyoxal (MG) is formed in plants during glycolysis and photosynthesis from dihydroxyacetone phosphate, which is catalyzed by triose phosphate isomerase to form glyceraldehyde 3-phosphate (Richard, 1993)**. The intermediate enediolate-P is formed in this reaction, which, after losing a phosphoryl group by beta-elimination, forms enol, which in turn is converted into MG. The reactions between the intermediate enediol and MG are non-enzymatic (blue arrows). The MG formed is eliminated by the sequential actions of glyoxalase I and II, in which the consumption and regeneration of reduced glutathione occurs.

Glyoxalases I and II (GLY-I and II) participate in the degradation pathway of methylglyoxal (MG), a toxic, mutagenic alpha-ketoaldehyde that may be lethal to cell functions (Ray et al., 1994; Kalapos, 2008). MG formation in plants begins with dihydroxyacetone phosphate (Figure 1) production in glycolysis and photosynthesis (Kaur et al., 2014). MG production increases considerably during stressful conditions, in which the glycolysis and photosynthesis pathways may become imbalanced, (Richard, 1993) and the MG levels may increase by two- to six-fold (Yadav et al., 2007). The accumulation of MG in plants increases the levels of intracellular oxidative stress due to the production of reactive oxygen species (Maeta et al., 2005; Kalapos, 2008), generates advanced glycation end-products (Thornalley, 2003), disables the mechanisms of the antioxidant defense system (Martins et al., 2001), and interferes with the cell division processes (Ray et al., 1994). MG degradation is initiated by a spontaneous reaction between MG and reduced glutathione (GSH) that forms hemithioacetal, which is then converted into S-D-lactoylglutathione in a reaction catalyzed by GLY-I (Figure 1). GLY-II releases D-lactate from S-D-lactoylglutathione and regenerates GSH.

Eleven genes encode GLY-I, and three encode GLY-II in rice (Mustafiz et al., 2011). The isoform expression patterns change depending on the plant tissue, stage of development, and environmental conditions applied. However, all expression patterns seem to have similar functions in MG degradation (Mustafiz et al., 2011). The genes *OsGLYI-3* and *OsGLYI10*, for example, are expressed only in seeds, while other isoforms are only present during specific stages of the embryo or endosperm. Each isoform may exhibit modified expression (induced or repressed) during stressful conditions. Experiments on two varieties of rice, one resistant to salt stress (Pokkali) and another sensitive to stress (IR64), showed different responses of different isoforms depending on the applied treatment. The OsGLYI-6 and OsGLYI-11 isoforms were increased when stress was applied. Both showed higher expression levels during salt, osmotic, and oxidative (adding H_2_O_2_ and MG) stresses (Mustafiz et al., 2011). These results support the conclusion that these two isoforms are important for the metabolism of MG but may also be important for protecting the cells against oxidative stress caused by ROS, given that GSH regeneration occurs during the MG metabolization process.

## Glutathione

The balance of the biosynthesis, transport, and degradation of glutathione is important in the defense against oxidative stress in plant cells in normal as well as in stressful situations (Noctor et al., 2012). GSH is continuously oxidized to GSSG (oxidized glutathione) and then regenerated by glutathione reductase (GR), which is dependent on NADPH. GSH is a key molecule in the cellular defense against oxidative damage caused by ROS, and new functions for this molecule are still being discovered. ROS preferably oxidizes GSH rather than molecules such as lipids, structural proteins, and nucleic acids, which prevents possible damage to these structures and their functions (Halliwell and Foyer, 1978). The role of GSH in antioxidant metabolism is well-discussed in the literature (Galant et al., 2011; Noctor et al., 2012).

**Figure 2:**
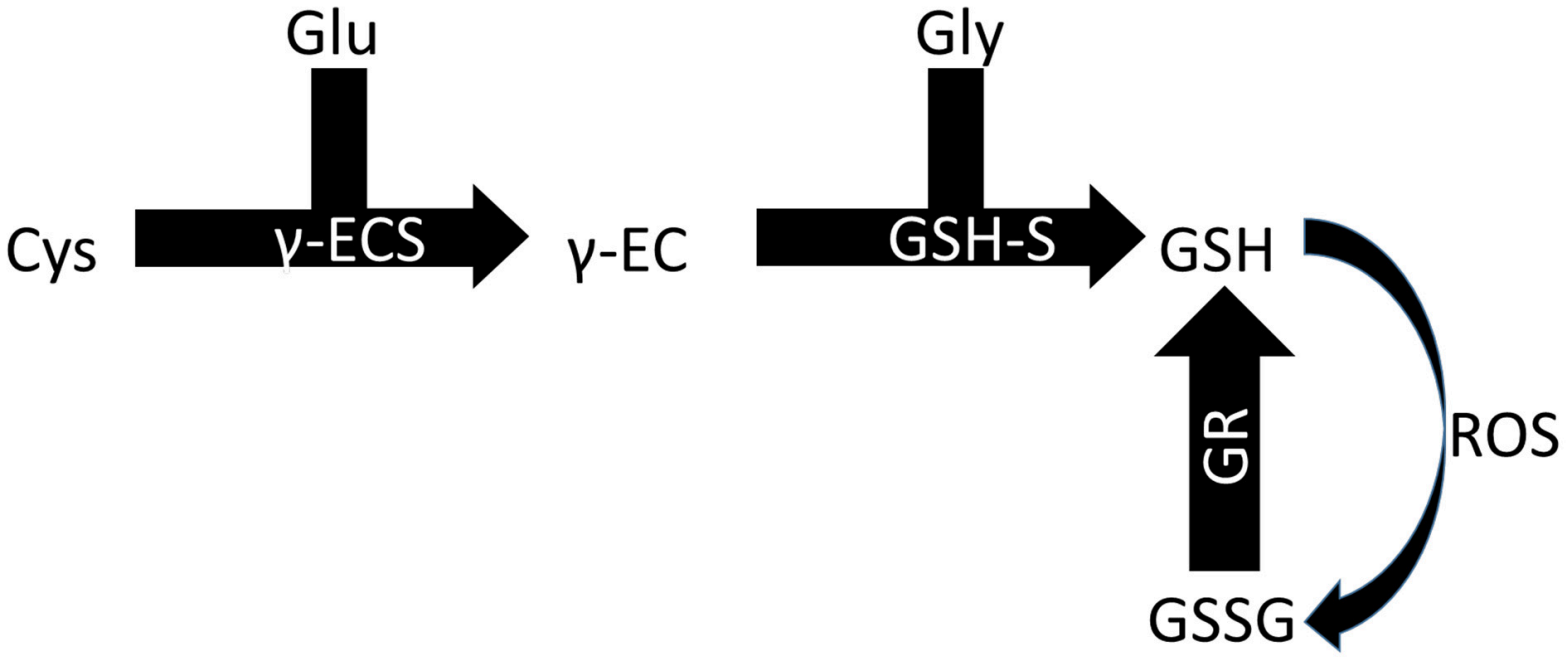
**Biosynthesis of GSH**. Glu, glutamine; Cys, cysteine; Gly, glycine; γ-ECS, gamma-EC synthase; GSH-S, glutathione synthetase; GR, glutathione reductase.

Two enzymes are responsible for synthesizing GSH in plants, each one encoded by a single gene, and GSH synthesis occurs in two ATP-dependent steps (Figure 2). The gene *GSH1* encodes the enzyme gamma-EC synthase (gamma-ECS), which is responsible for the first step of GSH synthesis, and the gene *GSH2* encodes the enzyme glutathione synthetase (GSH-S), which is responsible for the second step. In *Arabidopsis thaliana*, the first enzyme is likely exclusive to the chloroplast, while the second is present in the cytoplasm and chloroplast (Galant et al., 2011). Nevertheless, GSH is found in several cellular compartments, including the apoplast and phloem, indicating its ability to easily move within the intra- and extra-cellular environments (Noctor et al., 2012). The cytoplasm and chloroplasts are the cellular compartments that store most of the GSH in *A. thaliana* leaves, 50 and 30%, respectively (Queval et al., 2011).

## Ni may be a key factor for GSH homeostasis

Among the GLY-I isoforms studied in rice, OsGLY11.2 uses Ni as an activator (Mustafiz et al., 2014). Various stress types induce this isoform; therefore, suggesting (and determining) the importance of Ni for the redox balance of the cells during oxidative stress is possible. Other genes encoding GLY-I including *OsGLYI2, OsGLYI8, OsGLYI9.1, OsGLYI9.2*, and *OsGLYI12* do not exhibit altered expression in stressful situations. Among the three GLY-II genes, *OsGLYII-1* showed increased expression during the application of salt stress; *OsGLYII-2* showed high expression levels in all plant tissues (except seeds), and *OsGLYII-3* may provide tolerance during abiotic stress such as salt stress, heavy metals (Singla-Pareek et al., 2003, 2006, 2008), and MG accumulation (Yadav et al., 2007).

The gene expression levels and the activity of GLY-I may be increased by biotic and abiotic stresses, many of which have been related in the literature to increasing ROS production and, consequently, stimulation of the antioxidant metabolism for cellular protection (Mustafiz et al., 2011; Sharma et al., 2012; Wu et al., 2013; Kaur et al., 2014). Thus, the fact that Ni is an activator of a GLY-I suggests that it may play an important role in antioxidant metabolism.

Proving that Ni participates in the homeostasis of GSH in plant cells may not be simple, given that single and double mutants of *GSH1* and *GSH2* produce lethal phenotypes (Cairns et al., 2006; Noctor et al., 2012). In addition, the amount of MG in the leaves of certain plants (rice, Pennisetum, tobacco, and brassica) ranges from 30 to 75 μM under normal conditions (Yadav et al., 2005a), while overall, the total pool of GSH is nearly 20 times greater for plants (Noctor et al., 2012). The concentration of GSH is high in the leaves of plants growing under non-stressful conditions, where the GSH:GSSG ratio may be 20:1 (Mhamdi et al., 2010). In stressful situations, the amount of reduced molecules declines, and the GSH:GSSG ratio consequently changes (Mhamdi et al., 2010).

However, assays with double-mutant tobacco plants that overexpress GLY-I and GLY-II show that an effective relationship between the metabolism of MG and GSH may exist (Yadav et al., 2005b). When stressed with 200 mM NaCl, these mutants showed a GSH:GSSG ratio similar to that reported in unstressed wild-type plants. The activities of glutathione reductase, glutathione S-transferase, glutathione peroxidase, and ascorbate peroxidase were three- to four-fold higher in transgenic stressed plants than in the control unstressed plants, which clearly showed an associated effect between the metabolism of GSH and MG.

Potato plants overexpressing an ascorbic acid biosynthesis gene stressed with 200 mM NaCl maintained a higher GSH:GSSH ratio, which was followed by increased activity of antioxidant enzymes dependent on glutathione and glyoxalases, resulting in the inhibition of MG accumulation (Upadhyaya et al., 2011).

Therefore, the dependence between the two systems is clear. As this is a coupled system, the GLY-I dependence on Ni may play an additional role in the regeneration of GSH and therefore in homeostasis. Considering that MG is synthesized from dihydroxyacetone phosphate (generated mainly during glycolysis within the cytoplasm) and that the highest GSH concentration is also found in the cytoplasm, depending on the intensity of the stress MG production may be significant. In this context, Ni may be a key element for protecting plants against stressful conditions by decreasing the level of MG through the activity of glyoxalase as well as participating in the regulation of the GSH pool. Evidences exist and are strong, but new experiments will be necessary to prove that. Thus, the essentiality of Ni may be defined not only by urease activation, but also by modulating stress tolerance.

## Author contributions

All authors contributed to this perspective article.

### Conflict of interest statement

The authors declare that the research was conducted in the absence of any commercial or financial relationships that could be construed as a potential conflict of interest.
